# Production cost of autologous platelet rich plasma gel*

**DOI:** 10.1590/1518-8345.3265.3221

**Published:** 2019-12-05

**Authors:** Andrea Pinto Leite Ribeiro, Beatriz Guitton Renaud Baptista de Oliveira

**Affiliations:** 1 Fundação Osvaldo Cruz (FIOCRUZ), Instituto Nacional de Saúde da Mulher, da Criança e do Adolescente Fernandes Figueira, Rio de Janeiro, RJ, Brazil.; 2 Universidade Federal Fluminense, Escola de Enfermagem Aurora de Afonso Costa, Niterói, RJ, Brazil.; 3 Scholarship holder at the Coordenação de Aperfeiçoamento de Pessoal de Nível Superior (CAPES), Brazil.

**Keywords:** Platelet-Rich Plasma, Health Evaluation, Health Economics, Costs and Cost Analysis, Wound Healing, Nursing, Plasma Rico em Plaquetas, Avaliação em Saúde, Economia da Saúde, Cicatrização, Custos e Análise de Custo, Enfermagem, Plasma Rico en Plaquetas, Evaluación en Salud, Economía de La Salud, Costos y Análisis de Costo, Cicatrizácion de Heridas, Enfermería

## Abstract

**Objective:**

to estimate the direct cost of producing autologous platelet rich plasma gel.

**Method:**

an economic, prospective, longitudinal study with direct cost estimation, from the perspective of the Unified Health System, conducted in a university hospital in the state of Rio de Janeiro, over a period of 12 weeks. It was approved by the Ethics Committee of the School of Medicine. Direct observation of 18 participants was conducted. Material and human resources categories were analyzed for production costs.

**Results:**

the cost of producing platelet rich plasma gel was US $4.88 per session, for a total of US $5.16, when the material resources per unit were considered in the Unified Health System. The time to complete the procedure was approximately 22 minutes.

**Conclusion:**

the production of platelet rich plasma gel involves low cost material resources for both blood collection and preparation, enabling universal access to treatment. The procedure requires trained staff in an appropriate location; it is a safe and inexpensive technology.

## Abstract (pt)

**Objetivo:**

estimar o custo direto da produção do gel de Plasma Rico em Plaquetas autólogo.

**Método:**

estudo econômico, prospectivo, longitudinal, com estimativa do custo direto, cuja perspectiva foi do Sistema Único de Saúde, realizado em um hospital universitário, no estado do Rio de Janeiro, com horizonte temporal de 12 semanas. Aprovado pelo Comitê de Ética da Faculdade de Medicina. Realizada observação direta de 18 participantes. Na estimativa do custo da produção consideraram-se as categorias: recursos materiais e humanos.

**Resultados:**

o custo da produção do Gel de Plasma Rico em Plaquetas foi de US$4.88 dólares por sessão, e de US$5.16 dólares, considerando os recursos materiais em unidades que compõem o Sistema Único de Saúde. O tempo do procedimento foi de aproximadamente 22 minutos.

**Conclusão:**

conclui-se que a produção do gel de Plasma Rico em Plaquetas envolve recursos materiais de baixo custo tanto para coleta de sangue como para o preparo, possibilitando o acesso universal ao tratamento. O procedimento requer uma equipe treinada, em local adequado, sendo uma tecnologia segura e de baixo custo.

## Abstract (es)

**Objetivo:**

evaluar el costo directo de la producción del gel de Plasma Rico en Plaquetas Autólogo.

**Método:**

estudio económico, prospectivo, longitudinal, con estimativa de costo directo, cuja perspectiva fue el Sistema Único de Salud, realizado en un hospital universitario, en el estado de Rio de Janeiro, con un horizonte temporal de 12 semanas. Aprobado por el Comité de Ética de la Facultad de Medicina. Se realizó una observación directa de 18 participantes. En la evaluación de costos de la producción son consideradas las categorías: recursos materiales y recursos humanos.

**Resultados:**

el costo de la producción del Gel de Plasma Rico en Plaquetas fue de US$4.88 dólares por sesión y de US$5.16 dólares, considerando los recursos materiales en unidades que componen el Sistema Único de Salud. El tiempo de procedimiento fue de aproximadamente 22 minutos.

**Conclusión:**

se concluye que la producción del gel de Plasma Rico en Plaquetas utiliza recursos materiales de bajo costo, tanto para la colecta de sangre como para la preparación de esta, siendo posible proveer acceso universal al tratamiento. El procedimiento exige tener un grupo de profesionales capacitado y un local apropiado, ya que es una tecnología segura y de bajo costo.

## Introduction

Platelet rich plasma (PRP) is considered a promising technology in topical therapy, as it contributes to the wound healing process. The mechanism of action is related to the actions of: biomolecules, such as adhesive proteins, which promote cell interaction, hemostasis, coagulation and extracellular matrix composition; coagulation factors and associated proteins that produce thrombin; fibrinolytic factors and associated proteins that produce plasmin and vascular remodeling; and, proteases and antiproteases, which act on angiogenesis, vascular remodeling and coagulation control. Growth factors promote chemotaxis, cell proliferation and differentiation, and angiogenesis; chemokines, cytokines and others act to regulate angiogenesis and intracellular communication; and, antimicrobial proteins have bactericidal and fungicidal actions^(^ 1 ^)^.

Thus, the PRP acts in the various healing phases, promoting the shortening of the inflammatory phase by means of hemostasis, the provisional fibrin matrix, and the reduction of biofilm, promoting the formation of granulation tissue (chemotaxis, angiogenesis, and cell proliferation), epithelialization, keratinocyte proliferation and migration, and remodeling, with extracellular matrix synthesis^(^ 1 ^)^.

Platelet rich plasma is a product that originates from the centrifugation of whole blood, which is rich in growth factors and structural proteins that stimulate collagen and the extracellular matrix production that promote tissue repair, and which stimulate neovascularization and tissue regeneration^(^ 2 ^)^. It can be of an autologous nature, when the blood used for centrifugation comes from the patient himself; homologous, when the source is another patient; and heterologous, when it is derived from animal blood. The effectiveness of autologous, heterologous, and homologous PRP requires further studies^(^ 3 ^)^.

Studies performed in humans have shown the effectiveness of topical PRP in stimulating the healing of chronic wounds, such as diabetic ulcers, when compared with other antiseptic dressings for both healing and infection prevention^(^ 4 ^)^. Studies with venous ulcers have shown improved area reduction and a higher number of healed ulcers^(^ 5 ^-^ 8 ^)^.

Platelet rich plasma infiltrations have also been studied in clinical trials, such as: meniscus muscle repair with functional improvement after 18 weeks^(^ 9 ^)^; decrease of complications of primary total joint arthroplasty, with diminished bleeding and improved healing^(^ 10 ^)^; and, the use in knee osteoarthritis showed improvement of degeneration and quality of life^(^ 11 ^)^.

Autologous PRP, considered to be a bio stimulant, has also been used in dermatology for facial, neck, and hand rejuvenation due to its ease of application, lower risk of infection and allergies, in addition to its having hemostatic properties that reduce the possibility of bruising^(^ 12 ^-^ 14 ^)^. In addition to dermatological treatments, it has been used in areas such as androgenic alopecia in men and women, where it demonstrated an increase in the quantity and caliber of hair^(^ 15 ^)^. Thus, the use of PRP has grown significantly in topical therapies.

It can be prepared using commercial kits, a closed system, or by centrifugation and preparation with technical handling of the supernatant after centrifugation, by aspiration, in an open system.

Treatment with PRP has been considered experimental in Brazil, according to the Federal Council of Medicine Opinion No. 20/2011, due to the need for more scientific evidence to support its regulation^(^ 16 ^)^.

Given this, the National Health Surveillance Agency (ANVISA - Agência Nacional de Vigilância Sanitária) determined, in Technical Note No. 64/2015, that the processing of PRP for autologous purpose is allowed on an experimental basis, and that closed systems used in health facilities should be regulated by ANVISA^(^ 17 ^)^.

The Federal Council of Dentistry, in Resolution No. 158, of June 8, 2015, allows venipuncture and manipulation of PRP, in a closed system, by a qualified dentist or a health professional in conjunction with the dentist^(^ 18 ^)^.

Currently, scientific evidence studies such as controlled clinical trials are being developed to establish the effectiveness of PRP. But there are still few studies that assess the cost of producing PRP, either by autologous or homologous means. Studies that do evaluate the cost have considered all the treatment performed, without separately describing the cost of performing the technique^(^ 5 ^,^ 19 ^)^. Other studies with economic designs, analyze the cost of kits available at a mean cost of € 132.90 per kit per PRP session^(^ 20 ^)^ and US $450.00 per two sessions^(^ 21 ^)^. Both of these studies were conducted outside the Brazilian context, and with a different socioeconomic scenario.

Given the worldwide evidence of PRP use, and the possibility of obtaining and using PRP, this study aims to contribute with evidence on the cost of human resources, specifically nurses, in obtaining blood and preparing the PRP.

Resource allocation effectiveness has been the subject of policy discussions, as with health spending growth and limited resources; decision-making on resource allocation should be based on health technology assessment, as the health economy is based on opportunity cost, that is, resources applied to certain programs and technologies imply that others are not provided, so that their cost is not only represented by the resources spent on that technology, but also on the value of what is no longer being provided^(^ 22 ^)^. In this sense, cost analyses and economic assessments can provide decision-making inputs, contributing significantly to health policies^(^ 23 ^)^.

Cost analyses are the key steps in providing support for the development of economic assessments^(^ 24 ^)^, which involve comparing the cost between one or more alternatives, and the outcomes of interventions.

The objectives were to estimate the direct cost of producing autologous PRP gel, and to compare the cost of PRP production, considering the material cost in units of the Unified Health System (SUS).

## Method

This was an economic, prospective, longitudinal study, with direct cost estimation, whose setting was the SUS, conducted in a university hospital in the state of Rio de Janeiro, over a period of 12 weeks.

Direct costs are related to the resources consumed directly in treatment, health intervention, or care, such as: material and human resources, products, and services^(^ 22 ^)^.

This study is part of a macroproject, “Effectiveness and Cost of PRP as a topical therapy for venous ulcer patients”, approved by the Ethics Committee of the School of Medicine, under Opinion No. 1,378,184.

For production of autologous PRP gel, two steps were necessary: Step 1 - participant’s blood collection by venipuncture, with vacuum catheter; Step 2 - blood centrifugation to prepare the PRP by means of an adapted single centrifugation technique^(^ 25 ^)^ and activation using 10% calcium glyconate in the PRP gel.

To estimate the direct cost of production, the following categories were analyzed: material and human resources.

Data collection was performed from May of 2016 to December of 2017, using direct observation to identify and quantify the cost items (material resources) required to perform the procedure. The length of time required to perform the procedure was timed. The human resources assessed were nurses who had been trained to perform the procedure for at least one year.

For the production of the PRP gel, a fixed angle bench centrifuge with a capacity of 12 tubes (conical bottom) of 15 ml, with an adjustable speed of up to 4000 rpm, and a timer of 1 - 60 minutes was used. For each session, the material resources and the minimum quantity required to perform the procedure are described in Figure 1.

**Figure 1:** Material resources required for autologous platelet rich plasma gel production

**Material resources for production of autologous platelet rich plasma gel**			
Step 1 - Blood collection		Step 2 - Blood centrifugation	
Description	Minimum quantity	Description	Minimum quantity
Tubes containing 3.2% sodium citrate (5mL)	04 tubes	a Disposable pipette	01 u
Cotton balls	02 u	01 mL syringe	01 u
Alcohol 70%	02 ml	10% Calcium Glyconate Ampoule	01 u
19 or 21 vacuum catheter with disposable adapter	01 u	40x12 needle	01 u
Post-puncture hemostatic adhesive dressing	01 u	Single tube	01 tubo
Procedure glove	02 u	Procedure glove	02 u

The value assignment was performed through data collection, in July of 2018. With regard to the criterion for value assignment for material resources: Category A was the price established by the University Hospital (HU) electronic trading floor; Category B, human resources, considered the cost of the procedure, represented by the professional’s salary per hour, multiplied by the time consumed (in minutes) to accomplish steps 1 and 2, divided by sixty (minutes).

Figure 2 provides an overview of the steps, cost categories, and sources of information related to value assignment for cost calculation.

**Figure 2:** Summary of steps and cost categories, July of 2018

Step	Cost category	Measuring technique	Information sources
1 - Blood collection	Nursing human resources	Activity time (timed)	Ministry of Planning, Development and Management
2 - Blood centrifugation			
1 - Blood collection	Material resources	Direct observation	Electronic auction of the University Hospital. Permanent Committee on Standardization of Medical and Hospital Materials
2 - Blood centrifugation			

The costs corresponded to the remuneration of all levels of staff, with and without additional qualifications, in order to evaluate the minimum and maximum value, respectively and regardless of employment type, provided by the hospital and available on the website of the Ministry of Planning, Development and Management. The mean value was $ 0.17 per minute; the minimum was $0.13 and the maximum was $0.21 per minute.

The costing formula for each PRP gel production session per participant (C) involved the cost of two steps: Step 1 - blood collection, and, Step 2 - blood centrifugation. In each step, the costs of the material resources category (A) and human resources category (B) were summed, as follows:

The cost of the PRP Session = A + B costs (Step 1) + A + B cost (Step 2);

To compare the cost of the session with PRP gel in the HU with other SUS units, material prices were gathered from the price panel platform of the Ministry of Planning, Development and Management, and are presented as mean, minimum, and maximum prices.

## Results

The Step 1 cost, in dollars per day per participant was US $1.23. The human resources and material resources category showed equivalent costs, as it is a procedure that does not require much time to perform. The mean time in minutes for blood collection was 4.0 ± 0.8 minutes. Still, the cost of the human resources category was slightly higher, representing 54.2% of the total cost (Table 1).

**Table 1:** Cost, in US dollars, of Platelet Rich Plasma Gel according to steps and categories: material and human resources. Niteroi, RJ, Brazil, 2018

Cost of Platelet Rich Plasma Gel at University Hospital*							
	Step 1 - Blood collection			Step 2 - Blood centrifugation			PRP gel cost^†^
	Material resources	Human resources	Total	Material resources	Human resources	Total	
One session per participant	0.57	0.67	1.23	0.69	2.96	3.65	4.88
Six sessions (mean)	3.39	4.02	7.41	4.16	17.73	21.90	29.31
Standard deviation	0.19	0.81	0.95	0.16	1.26	1.31	1.64
Minimal	2.80	3.00	5.97	3.50	14.67	18.87	25.80
Maximal	3.80	6.50	10.30	4.20	20.67	24.87	32.23
Total cost for 18 participants	61.05	72.33	133.38	74.95	319.19	394.14	527.52

* Quoted on September 6, 2018, US $1.00 = R$ 4.14;

† PRP = Platelet Rich Plasma

In step 2, the dollars per day per participant costs were US $3.65, and the category that most impacted the total cost was human resources (81%), as the time for the supernatant centrifugation and aspiration process, with the addition of 10% Calcium Glyconate, was 17.9 ± 1.3 minutes.

Thus, the cost of producing PRP technology, in dollars, considering material resources (MR) and human resources (HR), was US $4.88 per PRP application session, totaling US $29.31 ± 1.63 for six sessions (Table 1). The time required from blood collection through PRP delivery was approximately 22 minutes.

Most of the total cost for a PRP session was related to human resources (85.8%). The minimum cost of six sessions was US $25.80, and the maximum was US $32.23. The total cost of six sessions for the 18 participants was US $527.52.

The variation in material costs of the blood collection and PRP preparation steps is due to variation in the amount of materials that was reduced in a participant who performed only five PRP applications, due to healing of an ulcer prior to the 12-week follow-up time. The item of material resources that varied the most was the amount of catheters, as two participants had difficult punctures, which also increased the time required for the procedure to be performed by the nurse.

The estimated cost of one session per PRP gel participant in the HU was US $4.88. The pricing panel platform is US $ 0.48 (minimum cost), US $ 5.16 (mean cost), and US $ 11.13 (maximum cost). The cost found in the HU is equivalent to the mean cost presented on the platform (Table 2).

**Table 2:** Cost, in dollars, of platelet rich plasma gel in the Unified Health System. Niterói, RJ, Brazil, 2018

Comparative Cost of Autologous Rich Plasma Gel Production*				
	HU^†^	Pricing Panel Platform		
n=18		Mean	Minimum	Maximum
One session per participant	4.88	5.16	3.48	11.13
Six sessions (mean)	29.31	30.94	20.87	66.75
Standard deviation	1.64	1.49	0.99	2.75
Minimum	25.80	27.09	18.25	57.58
Maximum	32.23	33.91	22.82	70.98
Total cost for 18 participants	527.52	556.88	375.60	1201.56

* Quoted on September 6, 2018, US $1.00 = R$ 4.14;

† PRP = Platelet Rich Plasma

As shown in Table 2, the cost of a six-session PRP protocol at HU (US $29.31) is close to the mean cost (US $ 30.94) of the other units that make up the SUS, the minimum cost (US $20.87) is 29.8% lower than the HU, and the maximum cost (US $66.75) is 127.7% higher than the HU, according to the Pricing Panel Platform.

## Discussion

The PRP production method is considered to be simple, although it requires a speed-regulating centrifuge, and training for careful handling^(^ 2 ^)^.

The PRP gel production in this study was autologous, using single centrifugation for topical use. There are several methods and processes for converting whole blood into a product (PRP) for topical application; all include blood centrifuging. Differences in the centrifugation process include velocity, acceleration, deceleration, angulation, and radius, as well as the types of platelet lysis activators that can be: calcium chloride with or without thrombin, batroxobin (a proteolytic enzyme that acts on plasma coagulation), thrombin, and freezing^(^ 26 ^)^.

Autologous PRP has high therapeutic potential and can be used in various formulations and in various fields of medicine and bioengineering. In 2012, more than 40 autologous platelet products were already available, with different characteristics in relation to platelet enrichment, presence of leukocytes, activator type, and final volume, making it difficult to compare results between studies^(^ 27 ^)^.

The closed system PRP preparation kit with centrifuge and preparation material for up to 100 PRP ranges from US $811.35 to US $929.95, on the market.

Commercialized systems enable the fractionation of plasma rich growth factor (PRGF-Endoret), where the vacuum suction is controlled in the fractionation tube. After separation of the PRP occurs, calcium chloride for platelet activation^(^ 28 ^)^, or ascorbic acid, thrombin and calcium chloride are added to centrifuged plasma for lysis and activation of PRP (Autologel™) ^(^ 28 ^)^.

A cross-sectional study conducted in Malaysia evaluated the cost of single-centrifuged autologous PRP compared to commercial kits. The findings showed that the PRP produced with this technique contained a significantly higher mean platelet and leukocyte count than whole blood, and no difference was found in the mean blood level leukocyte and platelet counts between the single centrifugation technique performed and commercial kits, demonstrating that PRP can be produced with clinically available material resources of similar quality to the commercial kit, at a cost of 29.02RM (Malaysian Ringgit) or (US $7.02), with a preparation time ranging from 25 to 30 minutes from collection to final product. In the other four commercial kits evaluated, the preparation time ranged from 15 to 20 minutes, and the costs ranged from 400.00 to 1600.00 RM^(^ 29 ^)^. The cost of the human resource involved for preparation and the cost of the centrifuge was not evaluated in this study.

Another study, conducted in the United States, shows that it is possible to train professionals to obtain PRP, via a single centrifuge cycle at 3200 RPM (1430G), for 10 minutes, with a material cost of less than US $10, with a similar time as is suggested by commercial kit manufacturers^(^ 30 ^)^. In these studies, although the cost of human resources and the centrifuge has not been accounted for, it is clear that the preparation of PRP without the commercial kit reduces the cost, enabling it to be used in the different types of treatment in which PRP has been shown to be effective, including within the SUS.

Moreover, a barrier remains for comparing cost studies, because many studies use materials and methods that are used with commercial kits, which increases the cost of materials and restricts treatment in the public system^(^ 30 ^)^.

In the Brazilian SUS, there is still no record of commercial kit purchases in the Price Panel Platform of the Ministry of Planning, which makes it difficult to compare costs.

Regarding the procedure, it is important to consider that competence in performing the procedure directly influences the cost of a product. Training is an important step, and should be performed by competent professionals, generating an interface of inter-professional education. This is especially true when it is observed that the cost of human resources is higher than the cost of material in the development of processes and products.

In this study, human resources accounted for 85.8% of the total cost of one PRP session per participant. In other studies, the cost of human resources represented the largest percentage of the cost of outpatient procedures, such as the treatment of venous ulcers with Carboxymethylcellulose gel^(^ 31 ^)^ and compressive therapy^(^ 32 ^)^.

Nursing activities are permanent sources of scientific and technological innovation, requiring skilled labor, and having a direct impact on the basis of organizational productivity, while being “socially committed to the public policies of the Unified Health System (SUS)”. This occurs through technological care, when: applying new ideas and adopting best practices; improving processes, care models and protocols, aimed at customer care and Administrative Technology, with management of operational processes which require knowledge and skills; using indicators, administrative processes, and cost management in care^(^ 33 ^)^.

Nurses are the professionals who have incorporated the practice of care the most, and their professional development has been permeated by the acquisition of fundamental theoretical and philosophical bases, incorporating technological innovations without losing the values, vision, and mission of their profession which means “giving attention to”, treating, respecting, and welcoming the human being and his/her needs”. Thus, the professional advancement of nurses is important, considering that this is a human resource which is committed to the sustainability of the health system^(^ 34 ^)^.

The lack of studies on the cost of PRP production conducted in the Brazilian context that could be used to compare the findings of this research was a limitation of this study.

## Conclusion

The cost of producing autologous PRP gel for topical use involves low-cost material resources for blood collection and blood centrifugation, totaling US $4.88 per session, and US $29.31 ± 1.63, for a six-session protocol, considering the nurse as the human resource.

The cost of a six-session PRP protocol at the HU (US $29.31) is close to the mean cost (US $30.94) of the other units that make up the SUS; the minimum cost (US $20.87) is 29.8% lower than the HU cost, and the maximum cost (US $66.75) is 127.7% higher than at the HU, according to the Pricing Panel Platform.

Therefore, this study provides evidence of the Brazilian cost of producing autologous PRP gel, from the perspective of the SUS. It is also concluded that the production of PRP should be performed by a trained professional, in an appropriate place, as it is a safe and inexpensive technology that can be developed by health professionals, especially nurses, for outpatient application in patients with chronic injuries.
